# The Role of microRNAs in the Diagnosis and Treatment of Pancreatic Adenocarcinoma

**DOI:** 10.3390/jcm5060059

**Published:** 2016-06-16

**Authors:** Maria Diab, Irfana Muqbil, Ramzi M. Mohammad, Asfar S. Azmi, Philip A. Philip

**Affiliations:** 1 Department of Internal Medicine, School of Medicine, Wayne State University, Detroit, MI 48201, USA; diab.maria@gmail.com; 2 Department of Oncology, Karmanos Cancer institute, Wayne State University, Detroit, MI 48201, USA; irfana.muqbil@wayne.edu (I.M.); mohammar@karmanos.org (R.M.M.); azmia@karmanos.org (A.S.A.)

**Keywords:** pancreatic ductal adenocarcinoma, micro-RNA, biology, diagnosis, therapy, prognosis

## Abstract

Pancreatic ductal adenocarcinoma (PDAC) remains a very challenging malignancy. Disease is diagnosed in an advanced stage in the vast majority of patients, and PDAC cells are often resistant to conventional cytotoxic drugs. Targeted therapies have made no progress in the management of this disease, unlike other cancers. microRNAs (miRs) are small non-coding RNAs that regulate the expression of multitude number of genes by targeting their 3′-UTR mRNA region. Aberrant expression of miRNAs has been linked to the development of various malignancies, including PDAC. In PDAC, a series of miRs have been defined as holding promise for early diagnostics, as indicators of therapy resistance, and even as markers for therapeutic response in patients. In this mini-review, we present an update on the various different miRs that have been defined in PDAC biology.

## 1. Introduction

Pancreatic cancer is the fourth leading cause of cancer-related deaths in the United States, with 53,070 new cases expected in 2016, of which 41,780 are expected to die from disease [1]. Surgery remains the only potentially curative treatment. However, a majority of patients present with non-resectable disease; only 15%–20% are surgical candidates at the time of diagnosis [2]. Surgery has an overall morbidity and mortality of 24% and 5.3%, respectively [3]. Tumor size less than 3 cm, negative surgical resection margins, well-differentiated histology and absence of lymph node involvement are favorable prognostic indicators [4]. Following a pancreaticoduodenectomy (Whipple procedure), the five-year survival rate is 25%–30% for node-negative [5] and 10% for node-positive disease [6]. This can be explained, in part, by the tumor’s high resistance to chemotherapy, as well as its propensity to recur and metastasize early, which may be related to the persistence of cancer stem cells (CSCs). Gemcitabine remains a commonly used drug in this disease [7]. Nab-paclitaxel has recently been shown to add to the benefit of gemcitabine in patients with favorable performance status [8]. The combination of fluorouracil, leucovorin, irinotecan, and oxalipatin (FOLFIRINOX) was also shown to be superior to gemcitabine, but, due to its side effect profile, it is reserved for patients with good performance [9]. More recently, monotherapy with S-1, an oral fluoropyrimidine derivative, demonstrated noninferiority to gemcitabine [10].

In light of the disappointing statistics in the prognosis of pancreatic ductal adenocarcinoma (PDAC), early detection of malignant and premalignant lesions is key. Unfortunately, no effective screening tool has been identified to date [11]. The tumors markers carcinoembryonic antigen (CEA) and carbohydrate antigen 19-9 (CA 19-9) are neither sensitive nor specific for screening but are used to follow known disease if they were initially elevated [12,13].

microRNAs (miRNA) are small (19–25 nucleotides) non-coding ribonucleic acids (RNAs) that interact with messenger RNA (mRNA) and serve as negative regulators of gene expression [14,15] by binding to imperfect complementary regions in the 3′ untranslated region of the target messenger RNA (mRNAs), inhibiting their translation or leading to their degradation. They have been shown to influence cell differentiation, proliferation, and apoptosis [16]. They represent only 3% of the human genome, but regulate 20%–30% of the protein coding genes [17,18]. They were first described in *C. elegans* in 1993 [19], and have a tissue-specific expression that is modified in a number of different conditions, including malignancy. They have been profiled in many different malignancies including breast [20], lung [21], and colorectal cancer [22] and differential expression was detected with those malignancies, all of which have made miRNAs promising biomarkers. The aim of this review is to present the evidence on the utility of miRNA in the diagnosis, treatment, and prognosis of PDAC.

## 2. microRNA in PDAC Biology

An understanding of the processes that govern the development of PDAC is crucial as it sheds light on potential biomarkers of early diagnosis and rational systemic therapeutic approaches. Multiple mutations in the evolution of PDAC are influenced by miRNAs, which serve as tumor promoters or suppressors by silencing or promoting of downstream pathways [23].

Activating mutations in *KRAS* are present in more than 90% of PDAC [24]. miRNA-96, 126, and 217, all of which target *KRAS*, were found to be downregulated in PDAC compared to other noncancerous, as well as normal, pancreatic tissues [25,26,27]. Furthermore, re-expression of miR-96 and 217 suppressed *KRAS* activity and resulted in reduced tumor migration and invasion, suggesting their role as tumor suppressors [26,27]. Additionally, miR-217 overexpression phosphorylated AKT levels, suggesting that miR-217 also influences downstream signaling involving cell survival and proliferation [27]. In another study, Kent *et al.* showed that RAS-responsive element-binding protein (RREB1) repressed the expression of miR-143/145 by binding to the promoter of the cluster [28]. Interestingly, oncogenic *KRAS* G12D mutations induce expression of RREB1 in PDAC to check the expression of miR-143/145 cluster. As the miR-143/145 cluster expression targets RREB1 protein to inhibit a feed forward circuit of *KRAS* signals through RREB1, the *KRAS* (G12D) mediated overexpression of RREB1 simultaneously represses the miR143/145 cluster expression, resulting in promotion of *KRAS* mediated signaling. Loss of expression of let-7 family miRNAs was described for the first time by Torrisani *et al.* [29]. Expression of let-7 suppressed *KRAS* expression and mitogen-activated protein kinase activation (MAPK), and inhibited cell proliferation but failed to hinder tumor progression [29].

Inactivation of *p53* occurs in 50%–75% of PDAC, predominantly through missense mutations in the TP53 tumor suppressor gene [30]. Several studies showed that mutant p53 regulates the transcription of certain miRNAs, and, subsequently, influence the expression of their target genes either by degrading their messenger RNA or by inhibiting their translation [31,32]. miR-15a, a known transcriptional target of *p53*, was shown to be downregulated in PDAC [33]. The overexpression of miR-15a downregulated WNT3A and FGF7, resulting in reduced proliferation and survival of pancreatic cancer cells [33]. p53 has also been shown to induce the expression of miR-200 and repress that of Zeb1 and Zeb2, both of which are known activators of epithelial to mesenchymal transformation (EMT) [34]. In chemoresistant pancreatic cancer cell lines, miR-200 family was downregulated, suggesting a deregulated p53 signaling in those cell lines [34]. Furthermore, upregulation of Zeb1 was associated with downregulation of the miR-200 family expression [35]. The overexpression of miR-200 family led to the downregulation of Jag1, a target of Zeb1 and a ligand of the Notch pathway [35]. p53 not only regulates the expression of certain miRs but also is in turn modulated by specific miRs. miR-491-5p inhibited the expression of both TP53 and Bcl-XL genes, as well as mitogenic signaling pathways, such as STAT3 and PI-3K/Akt, resulting in decreased cell proliferation and induction of apoptosis [36]. Furthermore, Neault showed that miR-137 targets KMD4A messenger RNA during Ras-induced senescence, a tumor suppressor response, and activates both p53 and retinoblastoma tumor suppressor pathways [37]. miR-137 levels were found to be significantly reduced in PDAC; restoring its expression inhibited proliferation and promoted senescence of pancreatic cancer cells [37].

Aberrations in the expression of the *p16* genes have been described in PDAC [38]. Also known as cyclin dependent kinase inhibitor 2A, p16 functions as a tumor suppressor gene by regulating cell cycle and cellular senescence. Studies have shown the inhibitory role of miR-10b and -24 on the expression of p16 in malignancies other than pancreatic cancer [39,40]. Both miR-10 and -24 were overexpressed in pancreatic cancer [41,42].

The TGFβ/SMAD pathway has been implicated in EMT. Through binding with their receptors, transforming growth factor β (TGFβ) isoforms transduce the phosphorylation of SMAD2 and SMAD3, which in turn bind to SMAD4 and translocate to the nucleus, where they regulate the transcription of target genes [43]. Other SMADs include SMAD-1, SMAD-5, and SMAD-8, and are collectively referred to as R-SMAD. On the other hand, SMAD-6 and SMAD-7 are negative regulators of R-SMADs and referred to as I-SMADs, or inhibitory SMADs [44]. While TGFβ acts as a tumor suppressant in normal cells by inhibiting cell growth, in cancer cells, the TGFβ/SMAD axis is modified resulting in impaired mediation of growth arrest [45]. Overexpression of the messenger RNAs encoding for TGFβ was observed PDAC and was associated with poor prognosis [46]. There is evidence suggesting that various microRNAs are regulated by the TGFβ/SMAD pathway, while others serve as regulators of that same pathway. The 130a/301a/454 microRNA family regulates TGFβ signaling through suppressing SMAD-4 expression by directly binding to its 3’UTR sequence [47]. This cluster was found to be upregulated in PDAC [48]. In another study, miR-421 and -483-3p promoted PDAC progression through directly regulating the tumor suppressor DPC4/SMAD4 [49,50]. Furthermore, aberrant expression of miR-146a on dendritic cells from PDAC patients was observed, and repression of SMAD-4 resulted in impaired differentiation as well as inhibition of antigen presenting function of dendritic cells, suggesting a role of microRNAs in modulating the immune response in PDAC patients through regulating TGFβ/SMAD signaling [51]. Overexpression of miR-192 was associated with a reduction in the expression of SMAD-interacting protein 1 (SIP1) [52]. Through direct suppression of SMAD2 and SMAD3, miR-323-3p inhibited TGFβ signaling, resulting in decreased cell motility and metastasis [53].

## 3. microRNA in PDAC Diagnosis

Accumulating evidence is showing that miRNA profiles are cell-specific and tumor-specific [54,55]. miRNAs have been so far isolated from the pancreatic tissue, pancreatic juices, bile, stool, blood, plasma, and sera of patients with pancreatic cancer [56]. Circulating miRNAs, specifically, have several exceptionally appealing characteristics: they are abundant, they are strongly resistant to degradation or modification compared to protein or carbohydrate-based tumor markers, their isolation is non-invasive and their amplification is technically easy and inexpensive [57,58]. Several miRNA profiles were observed to discriminate pancreatic cancer from benign pancreatic pathology and healthy samples. Circulating miRNA-483-3p levels are overexpressed in PDAC compared to intrapapillary mucinous neoplasms and healthy controls, and plasma levels of miR-483-3p differentiated PDAC from intraductal papillary mucinous neoplasm (IPMN) with a sensitivity (Sn) of 43.8%, similar to that of CA19-9 (45%) [59]. Elevated serum miR-200a and -200b levels were associated with silencing of SIP1 and overexpression of E-cadherin in patients with pancreatic cancer and chronic pancreatitis compared to healthy controls [60]. Serum miR-200a and -200b distinguished patients with PDAC from healthy controls with a Sn and specificity (Sp) of 84.4% and 87.5% for miR-200a and 71.1% and 96.9% for miR-200b, respectively [60].

Compared to traditionally used markers, serum miR-1290 distinguished patients with low-stage pancreatic cancer from controls better than CA19-9 did, and it was also found to influence pancreatic cancer cell invasion capability [61]. miR-16 and -196a independently discriminated pancreatic cancer patients from those with chronic pancreatitis or healthy controls. When CA 19-9 was added to the analysis, the discrimination was more sensitive and specific compared to microRNA panel or CA19-9 alone, with a Sn of 92% and Sp 95.6% for the discrimination of pancreatic cancer from healthy controls, and 88.4% and 96.3% for discriminating pancreatic cancer from chronic pancreatitis [62].

Specific alterations in miRNA expression are also noted in metastatic disease. Singh *et al.* showed at least a two-fold downregulation of miRNA-205 compared to nonmetastatic disease [63]. On the other hand, miR-146a was upregulated. Diagnostic kits profiling differentially expressed miRNAs were investigated to distinguish benign, premalignant, and malignant pancreatic lesions [64]. Szafranska *et al.* developed the first miR diagnostic, miRInform Pancreas, which utilized miR-196a and -217 to differentiate chronic pancreatitis from PDAC; their diagnostic Sn and Sp were 95% [64]. Lee *et al.* identified a panel of four miRs (miR-21-5p, 485-3p, 708-5p, and 375) that distinguished PDAC from IPMN with a Sn and Sp or 95% and 85%, respectively [65].

Table 1 and Table 2 list miRs that were shown to be upregulated and downregulated, respectively, in patients with pancreatic cancer, compared to benign pancreatic pathology and/or healthy samples.

## 4. microRNA in Therapy

### 4.1. Role of miRNAs in PDAC Therapy Resistance

The poor prognosis of pancreatic cancer is in part attributed to the high resistance rates to conventional chemotherapy. Accumulating evidence shows that most solid tumors are composed of two portions: the bulk and the cancer stem cell population. The latter survive the initial chemotherapy and utilize their self-renewal capabilities to regenerate a secondary population of tumor cells that is resistance to therapy. This inherent characteristic of CSCs might be controlled by specific miRNAs [63]. Jung *et al.* detected differentially expressed miRNAs in CSCs, including miR-99a, miR-100, miR-125b, miR-192, and miR-429 [95]. Certain alterations in miRNA expression are associated with chemoresistance. miRNA-200 family expression downregulation was observed in gemcitabine-resistant pancreatic cancer cells [96]. The mechanisms through which miRNAs induce chemoresistance have been elucidated in some studies. Hamada *et al.* showed that miR-365 induced chemoresistance through directly targeting the adaptor protein Src Homology 2 Domain Containing 1 (SHC1) and apoptosis-promoting protein BAX. It also upregulated S100P and Inhibitor of DNA binding 2, both of which are cancer-promoting molecules [97]. On the other hand, miRNA-34 regulated Notch signaling, leading to reduction in pancreatic CSC population [97]. Another study showed that miR-1246 expression induced chemoresistance through downregulating CCNG2 [98].

### 4.2. Potential of miRNAs as PDAC Therapeutics

As miRNAs regulate multiple gene expressions and signaling pathways, miRNA-based therapies are at an advantage over single-gene therapy, and, at least hypothetically, targeting miRNAs is expected to produce more effective anti-cancer activities. To that goal, multiple approaches have been utilized *in vitro* and *in vivo*, aiming for the downregulation of oncogenic miRNAs and/or the restoration of tumor suppressor ones. Approaches included introducing a miR antagonist or use of an miR mimic agent [55]. Transfecting pancreatic CSCs with a miR-200c mimic decreased colony formation, invasion and chemoresistance of pancreatic CSCs by regulating EMT [99]. Lu *et al.* reached similar results with transfection of miR-200a [100]. On the same note, transfecting gemcitabine-resistant pancreatic cells with miRNA-205 and miR-7 reduced the expression of TUBB3 and Pak-1, respectively, and reduced the CSC population [63]. Administering complexed micelles of gemcitabine and the tumor suppressor miRNA-205 achieved significant inhibition of tumor growth in a pancreatic tumor model; immuno-histochemical analysis showed decreased tumor cell proliferation and increased apoptosis [101]. Transfection efficiency was >90%. In another study, targeting miR-21 with lentiviral vectors inhibited cell proliferation [102]. Pancreatic stellate cells (PSCs) represent the precursor cells for cancer-associated fibroblasts in pancreatic tumor stroma [103]. Kuninty *et al.* showed that suppressing miR-199a and -214 in PSCs abolished the PSC-driven pro-tumor effects and resulted in decreased tumor cell growth [103].

Using treatment with the demethylating agent 5-Aza-2′-deoxycytidine (5-Aza-dC) and HDAC inhibitor vorinostat (SAHA), Nalls *et al.* restored the expression of miR-34, a transcriptional target of p53, which induced apoptosis and inhibited cell cycle progression and epithelial to mesenchymal transition [104]. Systemic intravenous delivery with miR-34a and miR-143/145 nanovectors inhibited the growth of MiaPsCa-2 subcutaneous xenografts in mouse models; this was displayed even in the orthotopic setting [105]. Treatment with a synthetic (fluorinated) curcumin analogue, CDF, led to the downregulation of miR-21, restoration of miR-200 and tumor suppressor PTEN, and the killing of the CSC population, resulting in suppressed tumor growth [106]. This was previously observed in the work of Ali *et al.*, as well as others [96,107,108,109,110,111]. Oral curcumin was well tolerated and showed some response in one phase II trial [112]. In another study, treatment with isoflavone or 3,3′-diindolylmethane (DIM) reversed the EMT, restored expression of the miRNA-200 family, and resensitized pancreatic cancer cells to gemcitabine [113].

Following miR expression patterns over the course of treatment provides a tool to monitor tumor burden, as well as the emergence of resistant strains of cancer cells, which would prompt modifying therapy [114]. In two studies, plasma levels of miR-18a and 221 dropped postoperatively in nine and eight patients, respectively [86,90]; furthermore, in one patient who had recurrence after surgery, miR-18a levels re-elevated with no similar change in the levels of CA19-9.

## 5. microRNAs as Prognostic Biomarkers

Evidence shows that certain miR profiles are associated with a more aggressive disease and worse survival. In a meta-analysis involving 1525 patients, overall and disease-free survivals were significantly shorter in patients with high tumoral miR-21 [115]. This was further shown in the work of Abue *et al.* [59]. Poor survival was also linked to high miR-155, 203, 222, and 10b, and low miR-34a levels [115]. Similarly, lower expression of miR-183 reduced survival compared to higher levels, and was significantly associated with tumor grade, metastasis, and TNM stage [116]. Overexpression of miR-1290 was also associated with worse outcomes [61].

## 6. Other Noncoding RNAs

Although miRNAs have gained a lot of praise as future biomarkers for PDAC, other less popular small noncoding RNAs (snRNAs), as well as long noncoding RNAs (lnRNAs), are also being studied as diagnostic and prognostic biomarkers. Circulating U2 snRNA identified PDAC from controls with high sensitivity and specificity [117]. Overexpression of lncRNAs HOTAIR, HULC, MALAT1, and PVT1 were observed in PDAC compared to non-cancerous controls, and was associated with more aggressive disease [118,119,120,121]. In another study, overexpression of lncRNA was associated with inhibition of cell proliferation [122].

## 7. Conclusions

Accumulating evidence supports the strong involvement of microRNAs in the pathogenesis of PDAC, highlighting their many different roles in the KRAS, p53, and TGFβ/SMAD pathways, among others. Whether it is their abundance, their resistance to degradation, the feasibility of isolating them noninvasively, or the ease of amplifying them, miRNAs represent appealing biomarkers that have so far been linked to the diagnosis, therapy, as well as the prognosis of PDAC. However, despite the many efforts that have occurred, a practical application to be used in the clinic is still lacking.

## Figures and Tables

**Table 1:** miRNAs upregulated in pancreatic ductal adenocarcinoma (PDAC) compared to benign pancreatic pathology and/or healthy pancreas.

miRNA	Source	Reference
miR-10a, miR-10b, miR-146a, miR-204, miR-372	PDAC tissue	[41]
miR-16, miR-21, miR-155, miR-181a, miR-181b, miR-196a, miR-210	plasma	[62]
miR-155, miR-181a, miR-181b, miR--181b-1, miR-181c, miR-181d, miR-21, miR-221	PDAC tissue	[66]
miR-196a, miR-196b, miR-203, miR-210, miR-222	PDAC tissue	[67]
miR-196a, miR-155, miR-143, miR-145, miR-223, miR-31	PDAC tissue	[68]
miR-196a, miR-221, miR-222, miR-15b, miR-95, miR-186, miR-190, miR-200b	PDAC tissue	[69]
miR-221, miR-181a, miR-181c, miR-155, miR-21, miR-100	PDAC tissue	[70]
miR-132, miR-212	PDAC tissue	[71]
miR-223, miR-143, miR-27a, miR-21, let-7i, miR-145, miR-142-5p, miR-142-3p, miR-10a, miR-150, miR-214, miR-107, miR-146b, miR-100, miR-23a, miR-199a-5p, miR-222, miR-155, miR-103, miR-221, miR34a, miR130a, miR-331-3p, miR-24, miR-505	PDAC tissue	[72]
miR-107, miR-103, miR-23a, miR-1207-5p, miR-125a-5p, miR-140-5p, miR-221, miR-143, miR-146, let-7, let-7d, let-7e, miR-145, miR-199b-3p, miR-199a-3p, miR-138-1, miR-92b, miR-181, miR-1246, miR-31, miR-155, miR-26a, miR-17, miR-23b, miR-24, miR-500, miR-331-3p, miR-939	PDAC tissue	[73]
miR-196a, miR-200a, miR-21, miR-27a, miR-146a	PDAC tissue	[74]
miR-155, miR-203, miR-210, miR-222	PDAC tissue	[75]
miR-21, miR-221, miR-100, miR-155, miR-181b, miR-196a	PDAC tissue	[76]
miR-21, miR-210, miR-221, miR-222, miR-155	PDAC tissue	[77]
miR-21, miR-196a	PDAC tissue	[78]
miR-21, miR-155	pancreatic juice	[79]
miR-205, miR-210, miR-492, miR-1247	pancreatic juice	[80]
miR-26b, miR-34a, miR-122, miR-126, miR-145, miR-150, miR-223, miR-505, miR-636, miR-885-5p	whole blood	[81]
miR-483-3p, miR-21	plasma	[59]
miR-21, 210, 155, 196a	plasma	[82]
miR-21	plasma	[83]
miR-210	plasma	[84]
miR-100a, miR-10	plasma	[85]
miR-18a	plasma	[86]
miR-182	plasma	[87]
miR-10b, miR-30c, miR-106b, miR-132, miR-155, miR-181a, miR-181b, miR-196a, miR-212	plasma	[88]
miR-642b, miR-885-5p, miR-22	plasma	[89]
miR-221	plasma	[90]
miR-200a, 200b	serum	[60]
miR-24, miR-134, miR-146a, miR-378, miR-484, miR-628-3p, miR-1290, miR-1825	serum	[61]
miR-6826-5p, mi-6757-5p, miR-miR-3131, miR-1343-3p,	serum	[91]
miR-20a, miR-21, miR-24, miR-25, miR-99a, miR-185, miR-191	serum	[92]
miR-10b, miR-30c, miR-106b, miR-155, miR-181a, miR-196a, miR-212	bile	[88]
miR-21, miR-155	stool	[93]

**Table 2:** miRNAs downregulated in PDAC compared to benign pancreatic pathology and/or healthy pancreas.

miRNA	Source	Reference
miR-148a, miR-148b, miR-375	PDAC tissue	[66]
miR-216, miR-217, miR-375	PDAC tissue	[67]
miR-96, miR-130b, miR-148a, miR-217, miR-375	PDAC tissue	[68]
miR-375	PDAC tissue	[69]
miR-30d, miR-381, miR-29c, miR-30a, miR-874, miR-324-3p, miR-33b, miR-30c-1, miR-139-3p, miR-887, miR-141, miR-575, miR-28-3p, miR-665, miR-494, miR- 617, miR-564, miR-217, miR-130b, miR-148a, miR-708, miR-648, miR-148b, miR-345, miR216a	PDAC tissue	[72]
miR-1254, miR-559, miR-1274a, let-7f-1	PDAC tissue	[73]
miR-217, miR-20a, miR-96	PDAC tissue	[74]
miR-216, miR-217	PDAC tissue	[75]
miR-31, miR-122, miR-145, miR-146a	PDAC tissue	[77]
miR-148a, miR-217	PDAC tissue	[78]
let-7d, miR-146a	plasma	[83]
miR-375	plasma	[90]
miR-6075, miR-4294, miR-6880-5p, miR-6799-5p, miR-125a-3p, miR-4530, miR-6836-3p, miR-4634, miR-7114-5p, miR-4476	serum	[91]
miR-492, miR-663a	serum	[94]
miR-216	stool	[93]
